# American Anesthesiology Residency Programs: Website Usability Analysis

**DOI:** 10.2196/38759

**Published:** 2022-10-20

**Authors:** Noah Seto, Jeffrey Beach, Joshua Calvano, Shu Lu, Shuhan He

**Affiliations:** 1 College of Osteopathic Medicine Rocky Vista University Parker, CO United States; 2 Department of Anesthesiology and Critical Care University of New Mexico Hospital Albuquerque, NM United States; 3 Department of Anesthesiology University of Kansas Medical Center Kansas City, KS United States; 4 Department of Anesthesia Corrigan Minehan Heart Center Massachusetts General Hospital Boston, MA United States; 5 Harvard Medical School Boston, MA United States; 6 Department of Medicine Harvard Medical School Boston, MA United States; 7 Lab of Computer Science Massachusetts General Hospital Boston, MA United States; 8 Center for Innovation in Digital HealthCare Massachusetts General Hospital Boston, MA United States

**Keywords:** medical student education, education in anesthesia, technology in education, quality improvement, communication

## Abstract

**Background:**

The Association of American Medical Colleges has recently issued recommendations for the upcoming 2022-2023 application cycle that residency programs should conduct all interviews for this upcoming application cycle over the web. In light of these recommendations, many students will have limited exposure to anesthesiology programs and will rely on information gleaned digitally. This change means that the aspects of program websites used to provide information, such as size, structure, location, requirements, and contact information, will be crucial in helping prospective residents decide where and how to apply in the future. An evaluation of website usability, which includes initial appearance along with factors that influence its ease of navigation and convenience of use, can thus be applied to anesthesiology residency websites. Areas of need can be targeted to increase web presence and provide effective pathways to exhibit the different attributes of their programs to future applicants.

**Objective:**

This study aimed to compile a list of US anesthesiology residency programs and their websites while objectively analyzing the websites using a formally published usability scoring system, as well as to identify positive and negative trends to offer areas of improvement among anesthesiology residency websites.

**Methods:**

We included only 114 US anesthesiology residency program websites in our sample set, since some websites we analyzed showed errors or inconclusive. Website usability was separated into 4 distinct categories for analysis based on methodology outlined in previous literature on both health care website usability and residency website usability. The 4 categories were Accessibility, Marketing, Content Quality, and Technology. Each website was then analyzed and scored based on key components highlighted within the 4 categories. The multiple factors were then graded using a percentage system to create a comprehensive score for each program.

**Results:**

The highest scoring category was Content Quality (mean 4.7, SD 2.48, SE 0.23). The lowest scoring category was Technology (mean 0.9, SD 0.38, SE 0.04).

**Conclusions:**

Through the application of a health care website usability framework, multiple anesthesiology residency programs were analyzed and scored in the areas of Accessibility, Marketing, Content Quality, and Technology, which allowed us to determine the effectiveness of the usability of these websites to convey information to their end user. Websites must communicate vital information, with usability at the forefront, to continue to grow, especially as the United States faces challenges due to the COVID-19 pandemic. Our recommendation is that anesthesiology programs should strive to improve website usability to increase the ease by which applicants can collect vital information about anesthesiology programs. A few proposed solutions include making changes such as decreasing error pages on websites, migrating away from using in-line cascading style sheets, and improving web page loading speeds to improve the Technology category.

## Introduction

### Background

Due to the changes that the coronavirus disease brought to the medical education landscape, medical students and residency programs have had to adapt to the rapidly shifting residency application process. This change was predominantly a result of limited in-person contact, which not only affected opportunities for audition rotations but also in-person interviews for this upcoming application cycle. For the current residency cycle of 2022-2023, the Society of Academic Associations of Anesthesiology and Perioperative Medicine and Association of Anesthesiology Core Program Directors recommended that all anesthesiology residency programs commit to web-based interviews and virtual visits for all applicants [1].

Based on how guidelines have changed in the past and continue to change regarding away rotations and virtual interviews, both medical students and anesthesiology residency programs will be placing greater emphasis on different resources in their decision-making process compared to previous years. There are some factors that will be difficult for programs to change, such as the current circumstances regarding the COVID-19 pandemic along with a program’s nonmodifiable elements (ie, city, program size, and patient population). Digitally, there are opportunities for programs to better showcase their strengths by optimizing program website usability. The purpose of this research was to inform administrators of anesthesiology residency programs on how to best reach a wide audience of applicants.

### Website Usability for Anesthesiology Residency Programs

Usability is not limited to a website’s appearance; it also incorporates factors of “user experience” such as understandability, layout, and the accuracy of information [2,3]. Previous research has examined website usability for library websites, e-commerce, government websites, and even mobile news apps [3-7]. Website usability has also been used to analyze health care websites such as those of children’s hospitals, digital health care centers, hospitals, and cancer centers [7-11]. Increased usability on websites is typically correlated with higher level of engagement by users. As a result, industries unrelated to health care have created regulated guidelines to measure usability in 4 different areas: Accessibility, Marketing, Content Quality, and Technology [11-14]. Health care websites have been facing increasing pressure to conform to industry standards of user experience [14-16].

Due to the increasing role of anesthesiology residency program websites in engaging potential applicants, usability is becoming more relevant. Residencies within the specialties of neurosurgery, dermatology, general surgery, diagnostic and interventional radiology, urology, physical medicine and rehabilitation, orthopedic surgery, otolaryngology, radiation oncology, vascular surgery, cardiothoracic surgery, and plastic surgery have all had their websites previously analyzed for content quality [16-27]. To date, we believe that no analysis of anesthesiology residency website usability has been completed. The International Organization for Standardization has defined usability as “the extent to which a system, product, or service can be used by specific users to achieve specified goals with effectiveness, efficiency, and satisfaction in a specified context of use” [28]. In our study, we are using the term website usability as outlined by Huerta et al [7,9,10]. Although this definition deviates from the typical definition used by web developers, it is a proxy for user-based usability testing; the definitions by Huerta et al [7,9,10] are the only ones that have been published specifically for health care website usability. Through this study, we propose that anesthesiology programs have placed less emphasis on website development metrics due to the rapid shift and pace of technological advancements. We hope that this paper will educate anesthesiology residencies on how to showcase themselves to applicants and better optimize their web presence. 

### Objectives

The primary goals of this study were to (1) compile a list of US anesthesiology residency programs and their websites while objectively analyzing the websites using a formally published usability scoring system; and (2) identify positive and negative trends to offer areas of improvement among anesthesiology residency websites.

## Methods

A cross-sectional usability audit of US anesthesiology residency websites was performed.

### Sample Selection

**Figure 1:**
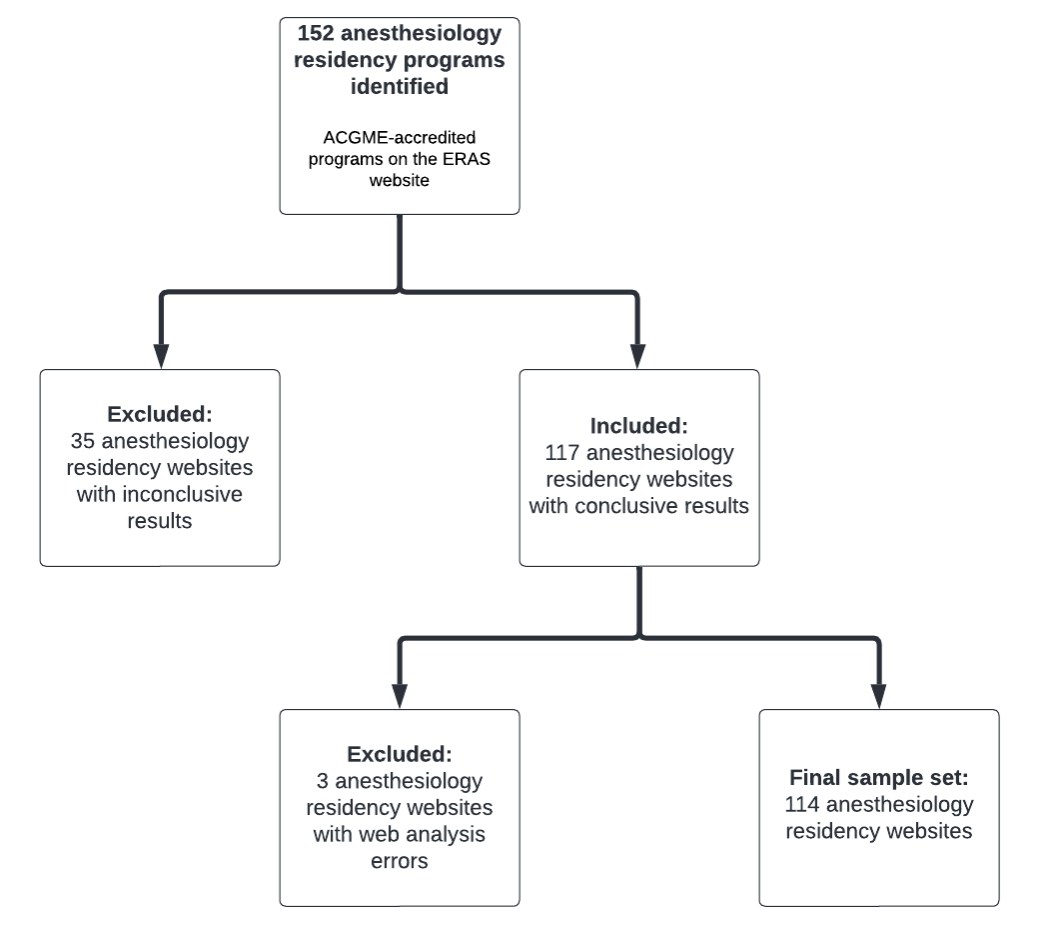
Sample selection criteria for anesthesiology residency websites. Inconclusive results are outlined in the Limitations section. ACGME: Accreditation Council for Graduate Medical Education; ERAS: Electronic Residency Application Service.

US anesthesiology residency programs were the target website population. We initially identified 152 anesthesia programs listed on the Electronic Residency Application Service that were accredited by the Accreditation Council for Graduate Medical Education (ACGME). Our sample set included only programs with their own primary domain or subdomain. Upon studying the limitations of the study by Fundingsland et al [27] due to program websites being subpages of a larger domain, we realized that our methodology could be modified to expand the inclusion criteria. We included a backslash to websites that were part of larger domains (ie, hospital or university) to limit the amount of non–residency-related content. This method ensured that all scores were accurate across all websites, regardless of the pages possibly being under a larger primary domain. If a website was analyzed and showed errors or had inconclusive results, they were excluded. An inconclusive result was defined as being unable to collect all data points with the tools. Commonly, this result was because of the inability to appropriately index a website by a web crawler due to blocking or website connection issues. Our final sample set included 114 US anesthesiology residency programs, with the selection process represented in Figure 1.

### Overview

Between March 12 and April 18, 2021, all data pertaining to website usability were acquired using tools assessing each programs’ website usability. Anesthesiology residency websites were ranked using the methodology and definitions previously defined by Calvano et al [8], Huerta et al [7,9,10], and Fundingsland et al [27]. Based on previous literature on website usability, 4 categories of website usability were both characterized and classified as follows:

1. Accessibility: the capability of users with minimal technology literacy to navigate web pages
2. Marketing: the ease with which websites can be accessed via search engines
3. Content Quality: the regularity of informational updates, readability, relevant content, and proper grammar
4. Technology: the excellence of the website’s coding, configuration of the website, and website loading speed [7-9]

### Analysis

All ACGME-accredited anesthesiology residency programs were compiled into a single database if they had a primary domain or subdomain. Each website was then scored, corresponding to the different tools defined by Calvano et al [8] and used by Fundingsland et al [27], as shown in Table S1 in Multimedia Appendix 1. The tools collected information on certain metrics, such as the number of missing texts, error pages, or missing files, that were quantified and then categorized. The main tool for extracting information was a web crawler. The web crawler analyzed websites and their respective subpages via URL to create an outline for metadata, errors, and improper links. Two authors (NS and JB) were trained by the expert author (JC) on both websites rating and data gathering. To ensure that the data collected were both replicable and exact, the raters also studied the instruction manuals related to each web tool. Each author was then assigned the tools they were the most confident in and collected the appropriate data for the tools used. When specifically examining factors dependent on internet speed, 2 separate tools were used with the results averaged to minimize the variability that could possibly be influenced by the authors’ internet connection. Each tool was run by either NS or JB on their respective computers. Both computers had the exact same specifications to minimize possible errors that could be introduced by differences in technology.

The selected usability tools were then applied and used to score each website. The results were then placed into 4 separate categories: (1) Accessibility, (2) Marketing, (3) Content Quality, and (4) Technology. Taking into account a variety of key factors relating to the 4 different categories, a General Usability score was then calculated. The different program websites were then ranked according to an Overall Usability score.

### Accessibility

Accessibility represents the ability of a website to engage with a varied population. Accessibility consists of the following components: readability, functionality, overall layout, and meta description. Readability was calculated by using tools that measured reading difficulty and approximated the grade level necessary to comprehend a web page. Functionality explores features that allow users of varying literacy proficiency to comprehend a website’s content. Accessibility encompasses the use of assistive technologies for those with visual impairments and those who would benefit from tools such as magnifiers or screen readers. A meta description is the brief outline produced when a website is found through a search engine. The results from the metrics used to analyze Accessibility were applied to an algorithmic scale to create a ranking list for program websites. Websites were ranked based on the estimated reading level needed for an appropriate understanding along with the ease of reading.

### Marketing

Marketing explores the difficulty of finding a particular website. This category was quantified by focusing on the website’s search engine results page (SERP). SERP is the placement of websites when searched through a search engine such as Google. As outlined in Table S1 in Multimedia Appendix 1, the analysis tool used was a combination of publicly available algorithms including Ahrefs (Ahrefs Pte Ltd) and Alexa ranking (Amazon).

### Content Quality

Content Quality examines the items and content on a website and a user’s ability to derive information from them. This category encompasses content such as the use of imagery/videos, metadata, analysis of text, and pertinence of text. When examining multimedia on web pages, the quality of images and the number of images were considered. Metadata serves as support for the presented content. Content Quality studies both the grammar and spelling of the written text. The pertinence of text evaluates the accuracy of information concerning certain topics. In our study, we evaluated anesthesiology residency websites based on their capability to present information that was grammatically correct along with the ease of deriving important information from each web page.

### Technology

Technology examines the technical performance of each anesthesiology residency website. Technology explores a website’s quality of technical design and performance. This category encompasses the comfort of use, user interface, server management, and server coding. User experience encompasses users’ emotions, preferences, and perception about a website, which can be influenced by the user interface. We were able to gauge the ease of use for the websites based on their ability to perform consistently across different devices, the general layout, and dissecting facets of HTML. This examination included ensuring that links on the websites lead to active pages while avoiding error pages. Server coding is the programming code implemented to ensure that websites run smoothly. Back-end design investigates that appropriate in-line cascading style sheets link to separate web pages. Loading time for websites is important not only for retaining users but also to ensure that the websites are accessible to new users.

### General Usability

The General Usability metric incorporates Accessibility, Marketing, Content Quality, and Technology. The score given is a summation of the applied percentages attributed to certain metrics outlined in previous research by Huerta et al [7,9,10]. It was created to quantify the overall quality of a health care website and produce a baseline for anesthesiology residencies to compare their own websites. 

### Overall Usability

Overall Usability was a calculation used to encompass the major and minor factors of the preceding 5 categories to create a rank list. The different factors in each category were then given a certain percentage according to their weight to create an all-encompassing ranking. The percentages were calculated from previously published research by Huerta et al [7,9,10]. A summation and description of the categories is shown in Table S1 in Multimedia Appendix 1.

## Results

From our initial set of 152 websites, 38 websites were removed. A majority (35/38, 92%) of the removed programs were due to limitations associated with the web crawler or security measures implemented on the websites to prevent crawling technology. A majority (35/38, 92%) of the problems were due to difficulties with the web crawler, because it occasionally lacked the ability to explore more than the opening web page. In other cases, there was not enough computing power for the web crawler to explore the thousands of subpages on a website. The remaining (n=114) anesthesiology residency websites were then scored based on our grading system by looking at their Accessibility, Marketing, Content Quality, Technology, and General Usability.

Content Quality was the highest average scoring category with a score of 4.7 (SD 2.48, SE 0.23). The overall lowest category was Technology, with a mean score of 0.9 (SD 0.38, SE 0.04). Accessibility had a mean score of 1.8 (SD 0.65, SE 0.06). Marketing had a mean score of 1.7 (SD 0.75, SE 0.07). Finally, General Usability had a mean score of 1.3 (SD 0.59, SE 0.06). Summary statistics of all categories are shown in Table 1.

**Table 1:** Anesthesiology residency websites: summary statistics from usability analysis.

Category	Mean (SD)	Standard error	Minimum	Maximum
Accessibility	1.8 (0.65)	0.06	0.00	6.47
Marketing	1.7 (0.75)	0.07	0.51	8.30
Content Quality	4.7 (2.48)	0.23	–2.23	20.48
Technology	0.9 (0.38)	0.04	0.67	4.91
General Usability	1.3 (0.59)	0.06	0.14	6.71

## Discussion

### Principal Findings

After studying the various aspects of website usability, Content Quality was the highest scoring category, whereas Technology was the lowest. Most anesthesiology programs also scored extremely low in General Usability. Anesthesiology residency programs with low General Usability scores indicate a shortcoming in comprehending the various components needed for a high-quality website. Superficially, websites might appear to be high quality; however, the necessary changes needed to truly optimize websites must be explored by conducting a usability analysis.

One way to improve Marketing is through search engine optimization (SEO). Many corporations use SEO to influence their SERP to market products. Applying SEO within anesthesiology programs can also increase their web presence and ability to share information.

According to our data in Content Quality, anesthesiology residency websites appeared to prioritize precise information about their programs. Thus, we can infer that anesthesiology residency websites are focused on the information they provide on the web.

The lowest ranked category on average was Technology, which indicated that anesthesiology residency programs had placed little emphasis on using new digital technology. The insufficient prioritization of technology manifests itself as websites that are seldom analyzed or have subpar server space. We acknowledge that the use of technology might be an area of greater difficulty for program administrators to address due to its technical nature, but it can be an area of growth within programs. By encouraging interprofessional collaboration between programs and their information technology departments, anesthesia programs would be better equipped for the rapidly changing technological world.

### Comparison With Prior Work

When comparing our study to related research that assessed the website usability of different specialties and residency programs, the areas for improvement appear to be uniform across all specialties. They all shared similar deficits and strengths in their websites, regardless of specialty. Although other studies assessed different residency websites using mechanisms or measurements that differed from our methodology, they all showed a need for the development of web presence for residency websites [18-27,29].

Our study used the same methodology that was previously performed by Calvano et al [8], which ranked the website usability of emergency medicine residencies. Previous research has enabled the authors to analyze usability trends related to health care, including children’s hospitals, digital health care centers, and hospitals [7-9]. Content Quality is still the highest scoring category when comparing previous usability research [7-9]. Similar to our study, Technology consistently appears as the lowest ranked category in previous studies [7,8]. Our results also show the Technology category having the lowest score, and this finding could be a result of a variety of problems such as missing files on web pages, slow loading times, and missing headers on web pages. One way to improve Technology is by advocating for greater collaboration between anesthesia programs and their information technology staff.

One point of divergence when comparing our study with previous findings appeared when examining children’s hospital websites. In children’s hospital websites, Accessibility was the lowest category instead of Technology [9]. Accessibility has also been ranked low in older studies [7,8]. Generally, Accessibility has tended to be lower in other studies but ranked second in our research. It could be presupposed that those who use residency websites already have the necessary educational background to appropriately comprehend the information presented; however, an objective of this study was to promote a common baseline for assessing websites. Health care has neglected the standardization of website analysis and now must double its efforts to be on par with other industries that have placed a greater importance on this area [11-13].

Health care is rapidly changing through the use of technology to not only improve care but also minimize expenses. As a result, usability is now becoming a vital part of evaluating health care websites, which also encompasses medical education and training. In addition, the COVID-19 pandemic and its unprecedented effects on the 2021-2022 application cycle and interview season highlight the importance of anesthesiology residency program websites.

### Limitations

We recognize the limitations of this study. The largest limitation is the decreased number of anesthesiology residency websites that were evaluated. Only 114 anesthesiology program websites out of the 152 ACGME-accredited anesthesia residencies met the inclusion criteria. With limited consumer-level analysis tools, multiple websites could not be properly analyzed for our study. Additionally, the web crawler was unable to examine many residency websites due to the random access memory demand from the tools and limited computing access by the data collectors.

Assessing the social media presence of the various programs proved to be a minor limitation. A large number of programs lacked direct links to their social media profiles. Consequently, we had to use the search engines within Facebook and Twitter. Further convoluting the process, the targeted pages often appeared lower on result pages, which introduced a concern about whether the correct, official social media pages were properly evaluated. This uncertainty underscored the importance of integrating appropriate and working social media links.

Another limitation was the measurement of each website’s loading time. Data were collected using a single computer and network to minimize confounding factors. The data were accumulated over 37 days, and the information collected might have changed since our initial examination of the websites.

Finally, the last limitation encountered was the small amount of research that applied this framework to health care websites. We recognize that website usability analyses are not universal, but we believe that further efforts should be focused on creating a database of health care website usability to increase relevance.

### Conclusion

The results of our study provide anesthesiology residencies a chance to critically examine their web presence and target areas that could use improvement. The average General Usability score of 1.3 indicates a need for overall improvement for anesthesiology residency program websites. Examining our data, anesthesiology programs do well with the Content Quality category, but there is room for improvement for the Technology category. Through this study, we advise anesthesiology residency programs to use recurring reviews of usability on their websites to ensure optimization in all categories of website usability.

## Appendix

Multimedia Appendix 1 Anesthesiology residency websites: factors that were assessed on anesthesia residency websites and the tools used to evaluate each website that were previously used in Calvano et al [1] and Fundingsland et al [27].

## Abbreviations

ACGME: Accreditation Council for Graduate Medical Education
SEO: search engine optimization
SERP: search engine results page
