# Supplementary material for: American Anesthesiology Residency Programs: Website Usability Analysis
Source: Interact J Med Res. 2022 Oct 20;11(2):e38759. doi: 10.2196/38759 (PMC9634514; doi:10.2196/38759)
Supplement: Multimedia Appendix 1 [file ijmr_v11i2e38759_app1.pdf]

Table S1. Anesthesiology residency websites: factors that were assessed on anesthesia residency websites and the tools used to evaluate each website.

**Assessment Factors:**	**Definition:**	**Accessibility**	**Content Quality**	**Marketing**	**Technology**	**General Usability**	**Overall Usability**	**Assessment Tools**	**Applied Formula**
**Alternative text**	*Missing image alternative text. This is used to offer a description of an image file contained on a webpage.*	15%		4%	4%	5%	6%	*Screaming Frog SEO Spider (Full)*	(Total Images - Missing Alt Text) / Total Images
**Amount of content**	*Assesses the amount of words present on an individual webpage.*	8%	33%	9%		5%	9%	*Screaming Frog SEO Spider (Full)*	Pages >600 words / Total pages
**Analytics**	*Utilizes a Google analytics service for tracking website traffic.*			4%	4%	3%	3%	*Browser Developer Tools (Free)*	Yes/No
**Broken Backlinks**	*Broken inbound backlinks. Backlinks are links from outside websites that link to the website of interest.*	8%		1%	8%	5%	4%	*AHREFS (Full)*	(Relative High - Broken backlinks) / Relative High
**Cascading Style Sheets (CSS)**	*Use of cascading style sheets (CSS) for meeting page design and styling standards.*	15%			8%	4%	5%	*Pingdom Tools (Free)*	Yes/No
**Domain age**	*The age of registered domain name.*			4%			1%	*GoDaddy WHOIS (Free)*	Absolute value
**Error page**	*Assesses for the number of error pages (4xx) within a given website.*			4%	8%	3%	3%	*Screaming Frog SEO Spider (Full)*	(Total Pages - Errors) / Total Pages
**Facebook**	*Number of Facebook likes for a company's social media page.*			8%		4%	3%	*Facebook (Free)*	Number of Likes / Relative High
**Headings**	*Number of missing H1 headers on a website.*	8%		4%	8%	5%	5%	*Screaming Frog SEO Spider (Full)*	(Total - X) / Total
**In-line CSS**	*Assesses for the use of embedded CSS throughout the site for additional page formatting.*	8%		1%	6%	5%	4%	*Pingdom Tools (Free)*	Yes/No
**Incoming Backlinks**	*Number of currently functioning backlinks.*			9%		5%	4%	*AHREFS (Full)*	X / Relative High
**Meta Data**	*Number of webpages missing meta descriptions.*	4%	13%	7%	4%	5%	6%	*Screaming Frog SEO Spider (Full)*	(Total - X) / Total
**Missing Files**	*Number of missing structured data files.*				8%	3%	2%	*Screaming Frog SEO Spider (Full)*	(Relative High - X) / Relative High

**Open Graph**	*Use of Facebook's Open Graph protocol*			4%	6%	4%	3%	*OpenGraphCheck (Free)*	Yes/No
**Popularity**	*Alexa Popularity Ranking*			9%		6%	4%	*Alexa Rankings (Free)*	(Relative High - X) / Relative High
**Printability**	*Whether the website utilizes printer-friendly CSS layouts.*				8%	5%	3%	*Pingdom Tools (Free)*	Yes/No
**Readability**	*Assesses the FK Reading Ease and Gunning Fog Index for reading difficulty and estimated grade level required for understanding.*	8%	33%	2%		5%	7%	*Readable (Free)*	FK 1/2: Absolute Value<br>GF 1/2:(Relative High - X) / Relative High
**Redirections**	*Number of redirections (3xx) within a website.*	6%			6%	1%	2%	*Screaming Frog SEO Spider (Full)*	(Total - X) / Total
**Social Interest**	*Total number of all social media interest (Twitter and Facebook factors combined).*			11%		5%	5%	*Facebook and Twitter (Free)*	Facebook Likes + Twitter Follows
**Speed**	*The average website speed across multiple assesment tools.*	6%		3%	15%	10%	7%	*Pingdom Tools and*<br>*Google Pagespeed Insights (Free)*	(Relative High - X) / Relative High
**Spelling**	*Percentage of spelling errors within a given website.*		20%				2%	*Readable (Full)*	100 - X / 100
**Twitter**	*Number of Twitter followers for a comany's social media page.*			7%		3%	3%	*Twitter (Free)*	X / Relative High
**URL format**	*Use of URL formats that submit to current SSH protocol.*	7%		7%	4%	4%	5%	*MOZ URL Structure (Free)*	Yes/No
**W3C compliance**	*Number of W3C compliance errors within a website.*	8%			8%	5%	4%	*W3C Markup Validation Service (Free)*	(Relative High - X) / Relative High
		**Note: Percentages do not add to 100 for rounding purposes.**						**Note: Full means that it was the paid, commercially available, version of the product. Free means that it was a free or open-**	**Note: "X" indicates the obtained value.**
